# Outcomes of Kidney Transplantation in Highly HLA-Sensitized Patients Treated with Intravenous Immuno-Globulin, Plasmapheresis and Rituximab: A Meta-Analysis

**DOI:** 10.3390/life14080998

**Published:** 2024-08-10

**Authors:** Deepak Chandramohan, Oluwadamilola Adisa, Devansh Patel, Erin Ware, Navya Eleti, Gaurav Agarwal

**Affiliations:** 1 Department of Internal Medicine/Nephrology, University of Alabama at Birmingham, Birmingham, AL 35233, USA; devanshhimanshupatel@uabmc.edu (D.P.); navyaeleti@uabmc.edu (N.E.); gauravagarwal@uabmc.edu (G.A.); 2 Department of Internal Medicine, Louisiana State University, Shreveport, LA 71103, USA; oadisa4@jh.edu (O.A.); erin.ware@lsuhs.edu (E.W.)

**Keywords:** kidney transplant, HLA desensitization, antibody-mediated rejection, anti-human leukocyte antigen, donor-specific anti-human leukocyte antibody

## Abstract

(1) Background: We aimed to investigate the outcomes of human leukocyte antigen (HLA)-incompatible transplantation for patients who received desensitization with intravenous immunoglobulins (IVIg), plasmapheresis, and rituximab. (2) Methods: A comprehensive search of multiple electronic databases to identify studies that utilized desensitization was conducted. The random-effects model was used to calculate the pooled rates and the 95% confidence interval (CI). (3) Results: A total of 1517 studies were initially identified. From these, 16 studies met the inclusion criteria, encompassing 459 patients, with a mean age of 45 years, of whom 40.8% were male. CDC crossmatch was positive in 68.3% (95% CI: 43.5–85.8; I2 87%), and 89.4% (95% CI: 53.4–98.4%; I2 89.8%) underwent living-donor transplantation. The 1-year graft survival pooled rate was 88.9% (95% CI: 84.8–92; I2 0%) and the 5-year graft survival rate was 86.1% (95% CI: 81.2–89.9; I2 0%). The 1-year patient survival rate was 94.2% (95% CI: 91–96.3; I2 0%), and the 5-year patient survival rate was 88.9% (95% CI: 83.5–92.7%; I2 7.7%). The rate of antibody-mediated rejection was 37.7% (95% CI: 25–52.3; I2 80.3%), and the rate of acute cell-mediated rejection was 15.1% (95% CI: 9.1–24; I2 55%). (4) Conclusions: Graft and patient survival are favorable in highly sensitized patients who undergo desensitization using IVIg, plasmapheresis, and rituximab for HLA-incompatible transplantation.

## 1. Introduction

Increased levels of human leukocyte antigen (HLA) sensitization occur in many patients due to several factors, such as prior blood transfusions, pregnancies, or transplantation, and this limits their chances of transplantation [1]. Anti-HLA donor-specific antibodies (DSAs) are a significant factor contributing to allograft rejections [2].

Since 2014, in the United States, the kidney allocation system has been modified to give increased priority to individuals who are highly sensitized to HLA in an effort to mitigate the challenges associated with HLA sensitization. Furthermore, the kidney paired donation program (KPD) in the USA and the kidney exchange programs (KEPs) available in European countries enable recipients with incompatible donors to undergo living-donor kidney transplants, provided there is a willing and approved living donor. However, compatible donors are not always available in the paired kidney exchange program, or such programs are not available in many countries [3,4]. Allograft survival through living-kidney HLA-incompatible transplantation (HLAi) has superior outcomes to dialysis, achieved after measures are taken to reduce the circulating antibodies through desensitization [5].

The approach to HLA-incompatible transplantation varies among transplant centers, but the overall aim is to improve the chances of successful kidney transplantation in patients with broad HLA sensitization and decrease the short- and long-term risk of rejection in such patients after transplantation. Plasmapheresis, intravenous immunoglobulin (IVIG), and B-lymphocyte antigen -CD 20 inhibitors have been the pioneer agents to be used for HLA desensitization and have been used for highly sensitized individuals undergoing a renal transplant. There is a lack of data from randomized controlled trials assessing the benefit of current desensitization protocols, and the ideal therapy remains unclear, particularly in terms of the long-term outcomes after desensitization. Nonetheless, the commonly used desensitization agents in many institutions include IVIg, plasmapheresis, and rituximab to decrease cell-mediated and antibody-mediated rejection in these patients who are highly HLA-sensitized [6].

During desensitization, these immunomodulators decrease the DSA by interrupting various pathways. IVIG works by regulating the immune response through multiple pathways [7]. This involves counteracting cytokines and antibodies, inhibiting T and B cells, increasing regulatory T cells, and inhibiting immune complex formation and dendritic cell activity [8]. Additionally, plasmaphereses work by removing immunoglobulins from the recipient’s serum. Plasma proteins, including albumin and coagulation factors, which are also removed in the process, are then replaced by albumin or fresh frozen plasma [6]. Rituximab, a monoclonal antibody against CD20, expressed on the surface of B cells and their progenitor cells, exerts its action by eliminating B cells in the peripheral blood and spleen. It also prevents their differentiation into plasma cells, although it does not directly affect plasma cells as they do not express CD20 [9].

The aim of our systematic review and meta-analysis was to evaluate allograft and patient outcomes after 1 and 5 years among HLA-incompatible transplantations in patients who received desensitization with IVIg, plasmapheresis, and rituximab. Additionally, this study also assessed the rates of rejection and infection following these desensitization techniques.

## 2. Materials and Methods

### 2.1. Data Sources and Search Strategy

A systematic review was conducted, as this method minimizes selection and interpretation bias. The meta-analysis component allows for quantitative data synthesis, since data on HLA desensitization are limited. Moreover, heterogeneity can be explored with meta-analysis methods. Hence, a systematic review and meta-analysis were conducted instead of a narrative review. The literature search was conducted by an experienced librarian who collaborated with the authors. A comprehensive search was conducted from study inception through August 2023 on the following electronic databases: PubMed, Embase, Web of Science, and Google Scholar. A combination of the following keywords was used in the search: “HLA desensitization” OR “hla antigens” OR “histocompatibility antigens class i” OR “histocompatibility antigens class ii” OR “immunologic desensitization” OR “desensitize” AND “kidney transplantation” OR “renal transplantation” OR “treatment”. Studies from study inception to August 2023 were included, and articles not written in English were excluded. Additional pertinent articles were discovered from the bibliographic sections of the articles of interest and were manually added. The search did not include gray literature. A detailed search strategy is shown in the Supplementary Materials.

### 2.2. Study Selection

The retrieved studies were independently screened for eligibility via abstract review by two authors (D.C. and A.O.). Following screening, full-text articles were reviewed, and studies were included based on inclusion and exclusion criteria. Any disagreements were resolved through consensus or discussion with another author (D.P.). The preferred reporting items for systematic review and meta-analysis guidelines were used to select the final articles [10]. The study protocol was registered in PROSPERO, an international database of systematic reviews, with registration number CRD42023425343.

The inclusion criteria were as follows: (1) patients ≥ 18 years old who underwent renal transplantation; (2) HLA desensitization using IVIg, plasmapheresis, and rituximab; and (3) studies published in English. The exclusion criteria were as follows: (1) pediatric studies; (2) studies pertaining to solid organ transplants other than kidney transplants; and (3) review articles, conference abstracts, and case reports.

### 2.3. Assessed Outcomes 

The primary outcome was graft and patient survival after 1 and 5 years. The secondary outcomes assessed were antibody-mediated rejection (AbMR) events, acute cell-mediated rejection events, transplant glomerulopathy, and infections. The infectious complications assessed were urinary tract infections (UTI), cytomegalovirus (CMV), and BK polyoma (BK) nephropathy. 

### 2.4. Data Extraction

The authors (D.C., D.P., and A.O.) extracted the data into a standardized form. The extracted data were verified by D.C. The author information, the country where the study was conducted, the total number of patients, demographic information, comorbidities, transplant wait times, re-transplants, induction agent used, post-transplant immunosuppression, follow-up period, rejections, infections, graft, and patient outcomes were recorded. 

### 2.5. Statistical Analysis

The categorical variables were expressed as percentages, and the continuous variables were expressed as mean ± standard deviation. A continuity correction factor was applied if the number of events in a study was reported to be zero. The studies were considered to be randomly chosen from a larger pool of studies, so we adopted the random-effects model [11]. The inverse-variance random-effects DerSimonian–Laird method was used to calculate the pooled rates, the mean estimates, and the 95% confidence intervals (CIs) [12,13].

Visualization of the statistical analyses was achieved by creating forest plots [14]. We assessed the heterogeneity using two methods. First, the Cochran Q statistic was applied to test the null hypothesis that all the studies included shared the same effect size. If this was true, then the expected value of Q would have exceeded the degrees of freedom (number of studies minus 1). The alpha level was set to 0.10 due to the limited statistical power. Subsequently, the I2 statistic was employed to quantify the percentage of variance in the effect sizes that was not solely attributable to sampling error once heterogeneity had been identified by the Q statistic. Different ranges of I2 values would thus indicate the degree of heterogeneity, with values <30%, 31% to 60%, 61% to 75%, and >75% being suggestive of low, moderate, substantial, and considerable heterogeneity [15].

The evaluation of the publication bias was performed by visual examination of the funnel plots alongside Egger’s test, and a *p*-value of <0.05 was considered significant. When publication bias was present, the Duval and Tweedie’s “Trim and Fill” method was used to assess the impact of bias [16,17]. All statistical analyses were conducted using the Comprehensive Meta-Analysis software, version 4 (Biostat, Englewood, NJ, USA) [18].

### 2.6. Quality Assessment and Risk of Bias

The modified Newcastle–Ottawa scale (NOS) was used to assess the risk of bias, since the studies included were primarily prospective studies. The studies were scored on study selection (representativeness of the exposed cohort, sample size, ascertainment of exposure, and ascertainment of outcomes at the start) and outcome (assessment of outcome, follow-up time, and adequacy of follow-up) [19]. Two authors (D.C. and A.P.) independently performed the scoring. The studies were evaluated for a maximum of 6 points, with a score of 5 suggesting a high quality and a score of <5 suggesting a low quality.

### 2.7. Ethical Approval and Consent to Participate

Ethical approval was not required for our meta-analysis because the data were already accessible to the public.

## 3. Results

### 3.1. Search Strategy Results

**Figure 1:**
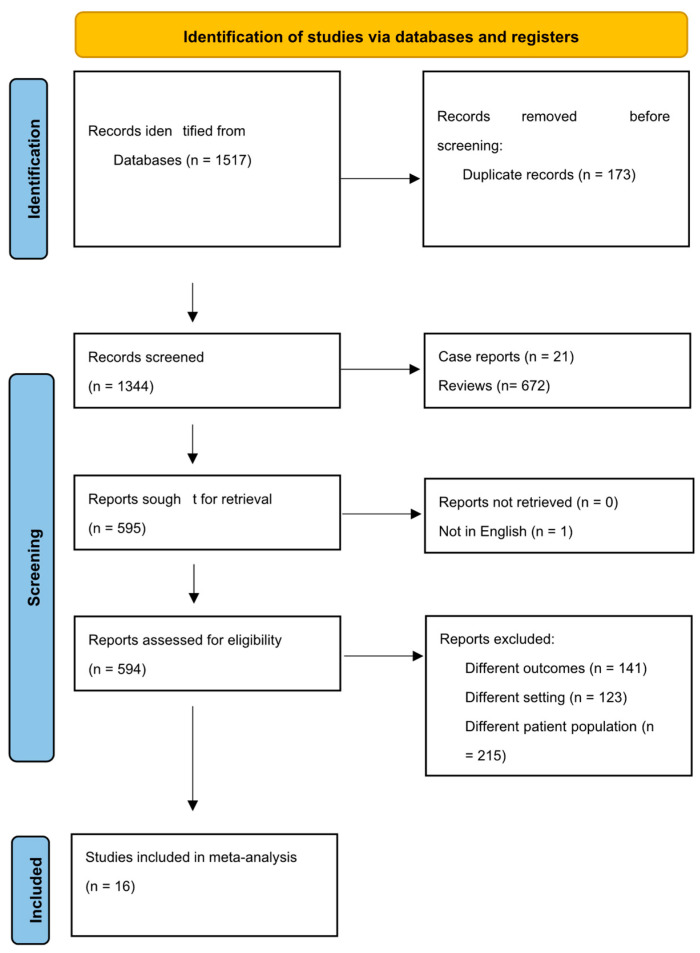
Study selection process according to the preferred reporting items for systematic review and meta-analysis (PRISMA) statement.

The initial search resulted in 1517 studies. Thereafter, a total of 1344 studies were retrieved after the duplicates were removed using a systematic review accelerator (SRA) [20]. Following screening, 595 articles were selected for full-text review. Finally, 16 studies were selected for the meta-analysis after applying the inclusion and exclusion criteria [2,21,22,23,24,25,26,27,28,29,30,31,32,33,34,35]. The study selection flowchart (preferred reporting methods for systematic review and meta-analysis) is shown in Figure 1.

### 3.2. Study Characteristics

There were fifteen retrospective cohort studies [2,21,22,23,24,25,27,28,29,30,31,32,33,34,35] and one prospective cohort study included in the meta-analysis [26]. In the study by Amrouche et al., the desensitization process was begun on the day of transplantation, while, in other studies, it began prior to transplantation. A high dose of IVIg of 2 g/kg was reported to have been used in five studies [2,25,27,28,36], and a low dose of 100 mg/kg was used in five studies [21,22,24,31,35]. One study by Stegall et al. reported using both low- and high-dose IVIg; 48 out of 61 patients in the study received low-dose IVIg, and the rest received high-dose IVIg [31]. The patients in the studies received between 1 and 21 sessions of plasmapheresis, and the rituximab dose was 375 mg/m^2^ in most of the studies.

Rabbit anti-thymocyte globulin was used as the induction agent in nine studies [2,21,22,27,30,31,32,33,35], whereas basiliximab was used in five studies [24,26,28,29,34]. The study by Reilla et al. reported using thymoglobulin in 42% recipients and basiliximab in 58% recipients [29]. Alemtuzumab was used only in one study by Kahwaji et al. [37]. Post-transplant immunosuppression was achieved with tacrolimus, mycophenolate mofetil, and prednisone in all studies except for Santos et al.’s study, where cyclosporin, azathioprine, and prednisone were used in 6% of the patients, while the rest were on tacrolimus, mycophenolate mofetil, and prednisone [30].

### 3.3. Patient Characteristics

A total of 459 patients underwent desensitization, 40% of whom were male while 59.2% were female. The mean age at transplant was 45 years (95% CI: 42.8–47.2; I2 73.9%). Among the patients who had undergone desensitization, the cause of ESRD was diabetes mellitus as a comorbidity in 16.9% of them (95% CI: 8–32.1; I2 77.9%), hypertension in 12.3% (95% CI: 6.6–21.6; I2 44.7), glomerulonephritis in 24.7% (95% CI: 14.8–38.3; I2 67.7%), and polycystic kidney disease in 8.5% (95% CI: 5.5–12.8; I2 0.0%). The mean dialysis vintage was 42.2 months (95% CI: 17.8–66.6; I2 97.5%). Notably, Santos et al. reported a dialysis vintage of 168 ± 97.2 months in their cohort [30]. Since this last study was an outlier with a significantly longer dialysis vintage, we also calculated the pooled dialysis vintage excluding Santos et al.’s study, and the pooled rate was 31.3 months (95% CI: 7.1–55.4; I2 97.8%). The patient characteristics are shown in Table 1.

Among the patients undergoing desensitization, living donors comprised 89.4% (95% CI: 53.4–98.4; I2 89.82%) of the cohort, and 52.0% (95% CI: 39.6–64.1; I2 67.3%) of the patients were undergoing re-transplantation. The mean panel-reactive antibody (PRA) was 57.7 (95% CI: 42.7–72.8; I2 96.7%). The complement-mediated cytotoxicity (CDC) crossmatch was positive in 68.3% of the subjects (95% CI: 43.5–85.8; I2 87%). The mean follow-up period was 41.7 months (95% CI: 26.7–56.6; I2 98.1%). The outcomes of the included studies are shown in Table 2.

### 3.4. Outcomes

**Figure 2:**
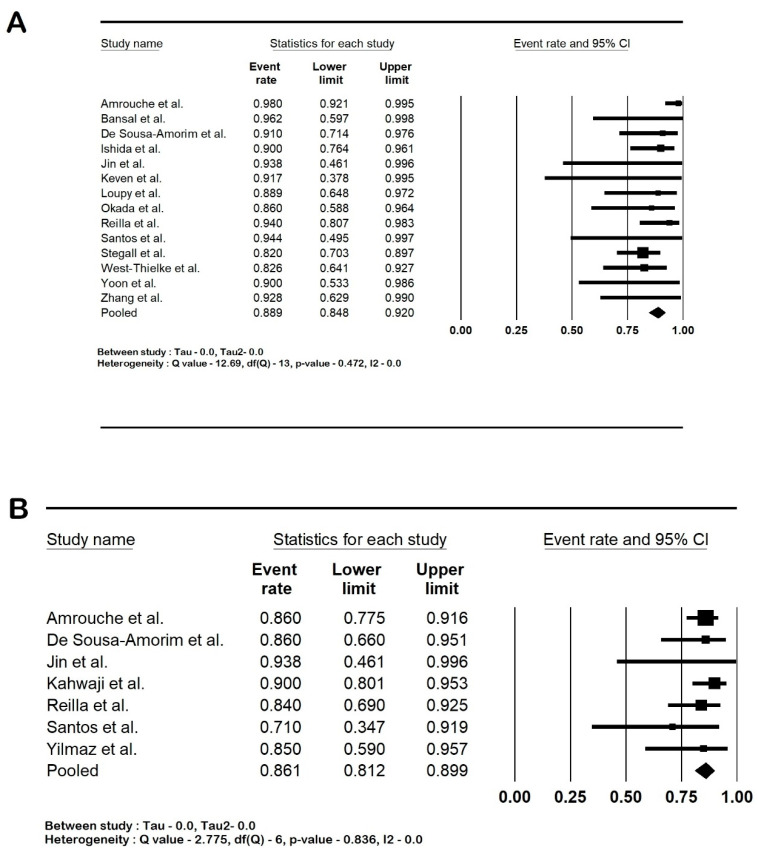
Forest plots of graft outcomes (death-censored): (**A**). 1-year graft survival and (**B**). 5-year graft survival Amrouche et al. [2], Bansal et al. [21], De Sousa-Amorim et al. [22], Ishida et al. [23], Jin et al. [24], Kahwaji et al. [25], Keven et al. [26], Loupy et al. [27], Okada et al. [28], Reilla et al. [29], Santos et al. [30], Stegall et al. [31], West-Thielke et al. [32], Yilmaz et al. [33], Yoon et al. [34] and Zhang et al. [35]. Abbreviation: CI, confidence interval.

**Figure 3:**
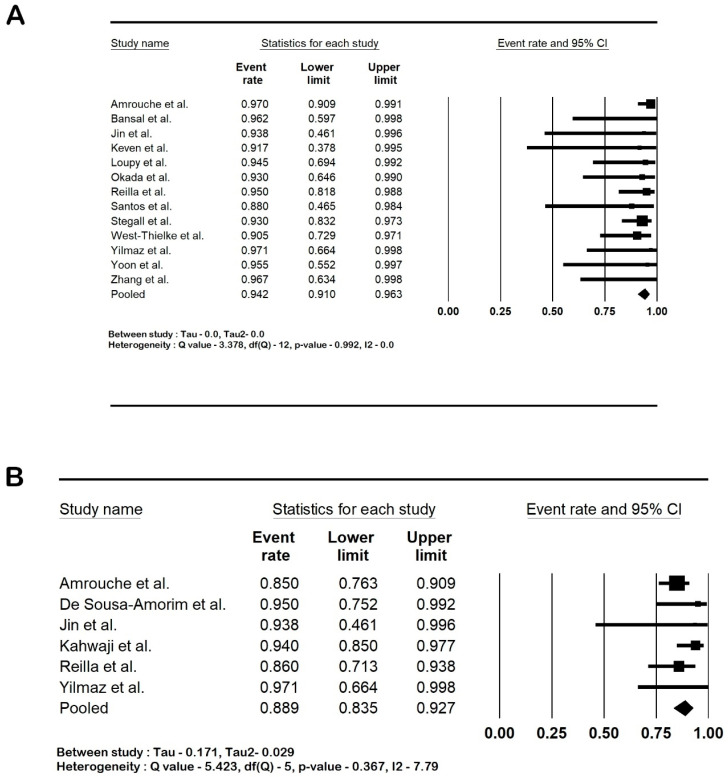
Forest plots of patient outcomes: (**A**). 1-year patient survival and (**B**). 5-year patient survival Amrouche et al. [2], Bansal et al. [21], De Sousa-Amorim et al. [22], Jin et al. [24], Kahwaji et al. [25], Keven et al. [26], Loupy et al. [27], Okada et al. [28], Reilla et al. [29], Santos et al. [30], Stegall et al. [31], West-Thielke et al. [32], Yilmaz et al. [33], Yoon et al. [34] and Zhang et al. [35]. Abbreviation: CI, confidence interval.

The 1-year death-censored graft survival rate was 88.9% (95% CI: 84.8–92; I2 0%), and the 5-year death-censored graft survival rate was 86.1% (95% CI: 81.2–89.9; I2 0%). The 1-year patient survival rate was 94.2% (95% CI: 91–96.3; I2 0%), and the 5-year patient survival rate was 88.9% (95% CI: 83.5–92.7%; I2 7.7%). The forest plots are shown in Figure 2 and Figure 3. 

**Figure 4:**
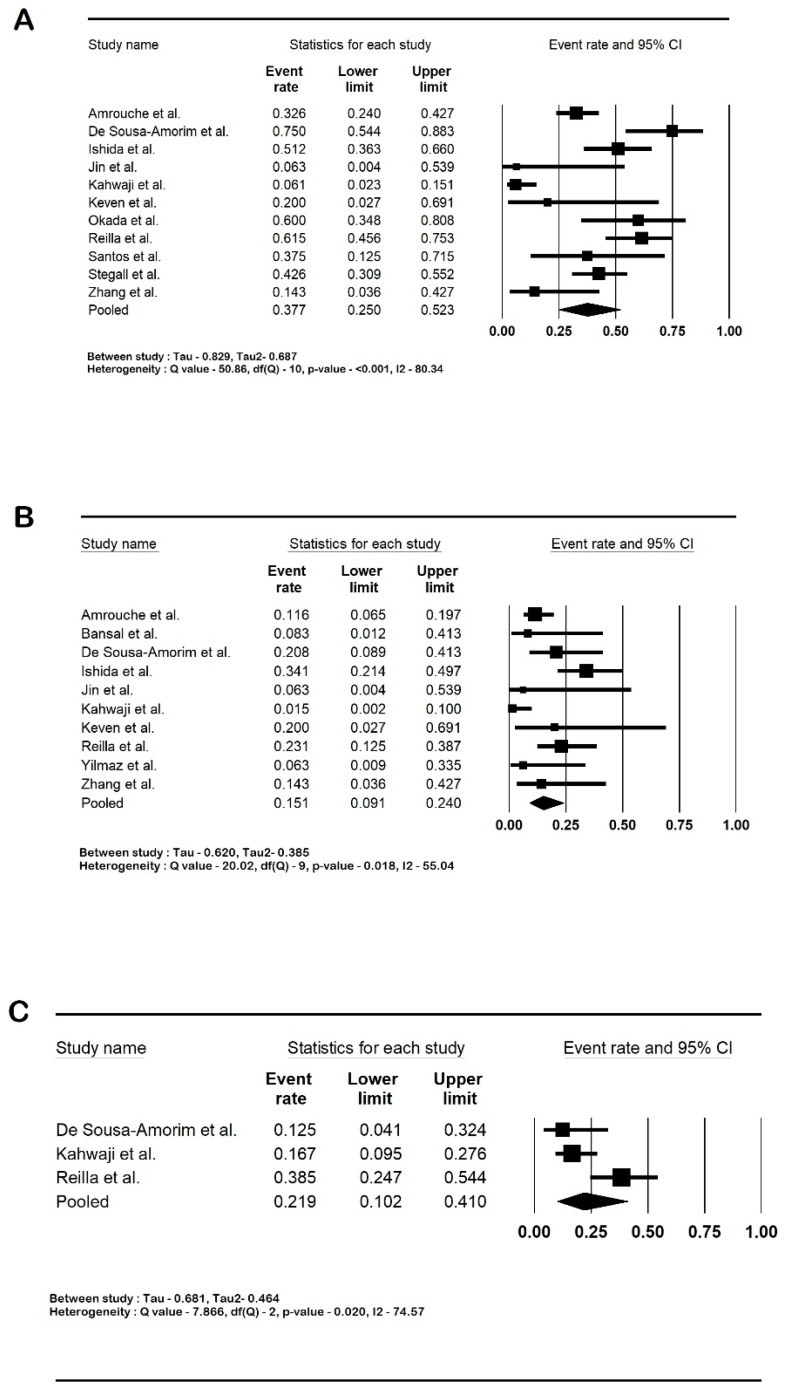
Forest plots: (**A**). antibody-mediated rejection; (**B**). acute cell-mediated rejection; and (**C**). transplant glomerulopathy. Amrouche et al. [2], Bansal et al. [21], De Sousa-Amorim et al. [22], Ishida et al. [23], Jin et al. [24], Kahwaji et al. [25], Keven et al. [26], Okada et al. [28], Reilla et al. [29], Santos et al. [30], Stegall et al. [31], Yilmaz et al. [33] and Zhang et al. [35]. Abbreviation: CI, confidence interval.

All the studies reported that the post-transplant biopsies had been conducted as per their institution’s protocol. The rate of antibody-mediated rejection was 37.7% (95% CI: 25–52.3; I2 80.3%), and the rate of acute cell-mediated rejection was 15.1% (95% CI: 9.1–24; I2 55%). Transplant glomerulopathy was present in 21.9% of the cases (95% CI: 10.2–41; I2 74.57%). The forest plots are shown in Figure 4.

**Figure 5:**
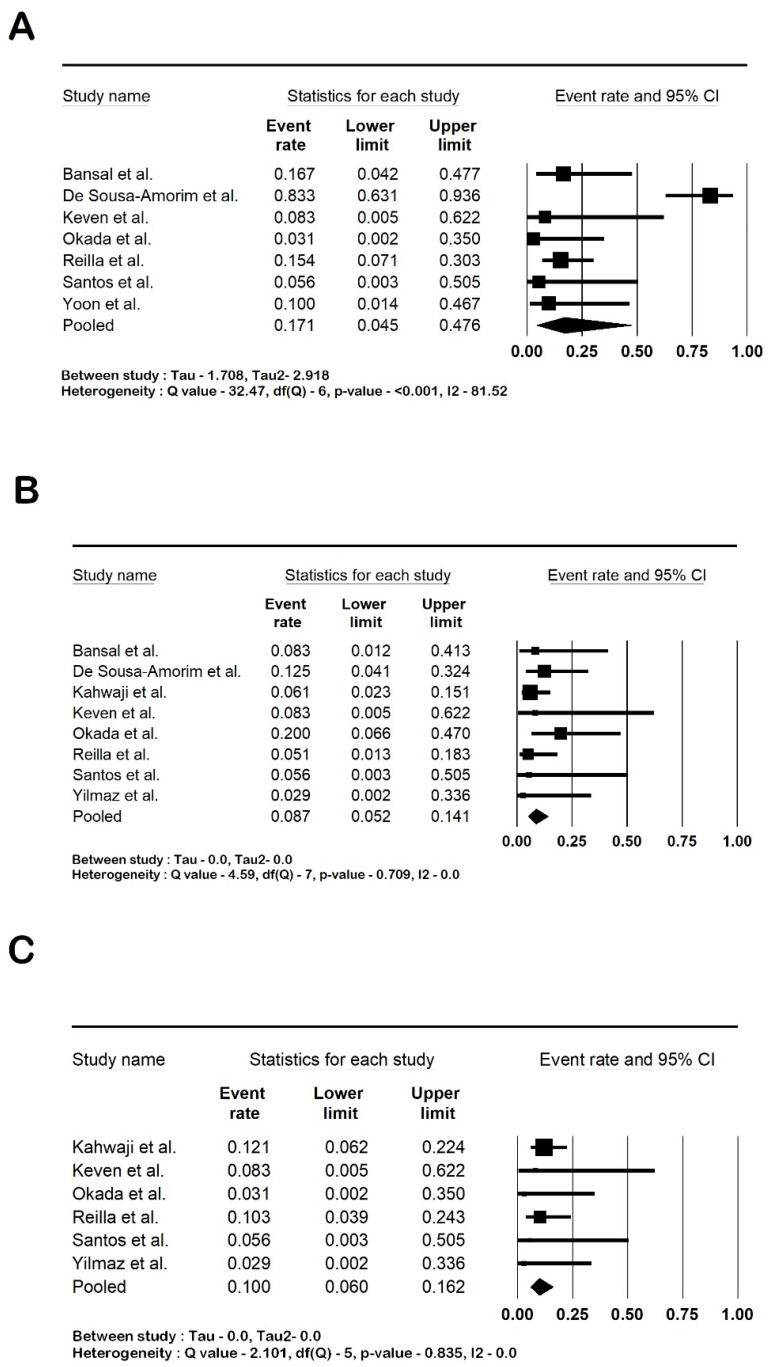
Forest plots of infections: (**A**). urinary tract infections (UTI); (**B**). cytomegalovirus infections (CMV); and (**C**). BK polyoma nephropathy. Bansal et al. [21], De Sousa-Amorim et al. [22], Kahwaji et al. [25], Keven et al. [26], Okada et al. [28], Reilla et al. [29], Santos et al. [30], Yilmaz et al. [33] and Yoon et al. [34]. Abbreviation: CI, confidence interval.

Among the infections reported, UTI was present in 17.1% of the patients (95% CI: 4.5–47.6; I2 81.5%), CMV infections in 8.7% (95% CI: 5.2–14.1; I2 0%), and BK nephropathy in 10% (95% CI: 6–16.2; I2 0). The forest plots are shown in Figure 5. Table 3 summarizes the pooled outcomes. A further analysis using a control arm was not carried out as the studies with controls had different definitions for the controls. 

### 3.5. Quality Assessment and Risk of Bias

The studies scored between 4.5 and 6.5 points on the NOS. A total of 11 studies were of a high quality [2,21,22,23,24,25,27,28,29,31,32], and the others were of a medium quality. Table 4 shows the quality assessment results and the risk of bias scoring.

### 3.6. Heterogeneity

Both the Q statistic and the I2 statistics were used to assess heterogeneity. When the Q statistic detected the presence of heterogeneity in an analysis, we used the I^2^ statistics to determine its degree. We concluded that the degree of heterogeneity was small during estimations of graft, patient survival, CMV infections, BK viremia, and BK nephropathy. Heterogeneity was considerable in the other effect sizes estimated, as they exceeded 75%. 

### 3.7. Sensitivity Analysis

**Figure 6:**
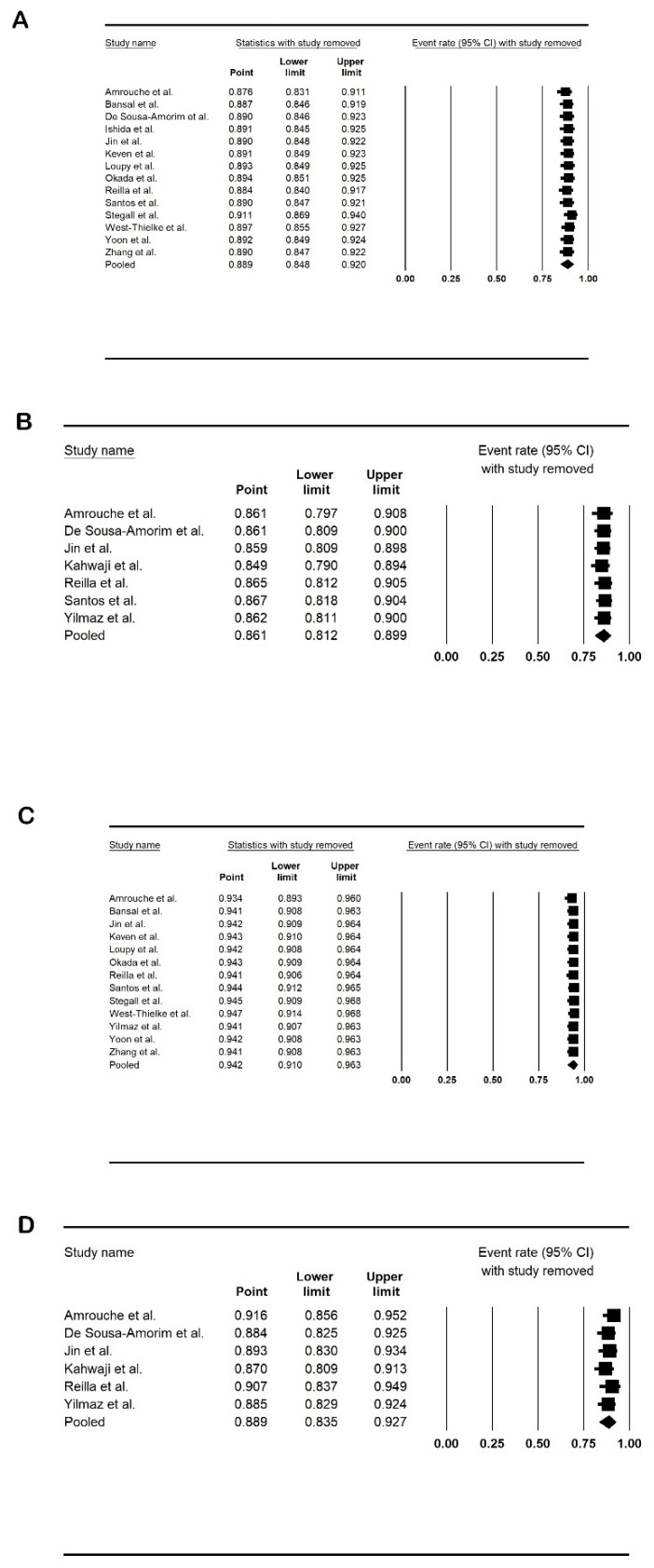
Forest plots of sensitivity analysis: (**A**). 1-year graft survival; (**B**). 5-year graft survival; (**C**). 1-year patient survival; and (**D**). 5-year patient survival. Amrouche et al. [2], Bansal et al. [21], De Sousa-Amorim et al. [22], Ishida et al. [23], Jin et al. [24], Kahwaji et al. [25], Keven et al. [26], Loupy et al. [27], Okada et al. [28], Reilla et al. [29], Santos et al. [30], Stegall et al. [31], West-Thielke et al. [32], Yilmaz et al. [33], Yoon et al. [34] and Zhang et al. [35].

A sensitivity analysis was performed to ascertain whether an individual study exerted a dominant effect on the effect size. Each study was systematically excluded, and the effect size was assessed to observe the impact of the excluded study on the summary estimate. Except for the pooled duration of dialysis, we did not find that a single study influenced the summary effect sizes. Figure 6 shows the sensitivity analysis. 

### 3.8. Publication Bias

**Figure 7:**
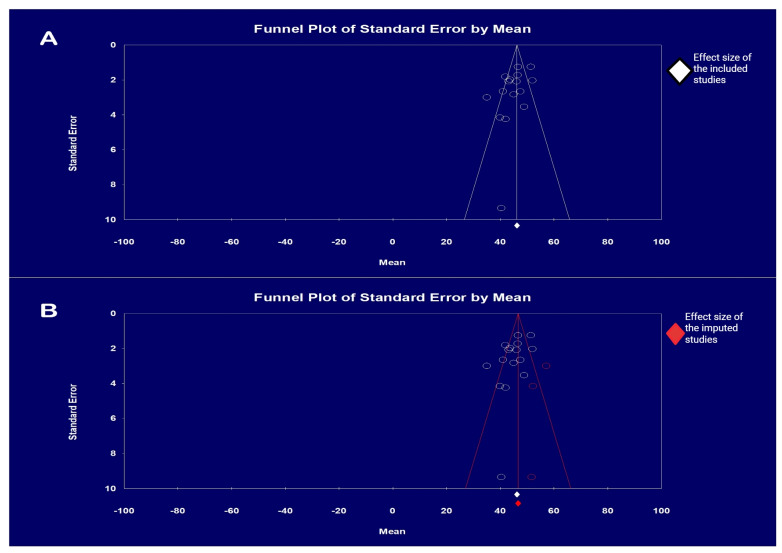
Funnel plots for the analysis of publication bias: (**A**). only the included studies, with Egger’s test for a regression intercept giving a one-tailed *p*-value of 0.029, indicating evidence of publication bias (the intercept (B0) is −2.349, 95% confidence interval (−4.80, 0.102), with t = 2.05, df = 14, and the two-tailed *p*-value is 0.059); (**B**). both the included and imputed studies by the “Trim and Fill” method, whereby, with the imputed studies, the effect size did not differ significantly from the previous effect size.

Our analysis of publication bias by visual inspection showed a potential publication bias due to the presence of asymmetry. Therefore, Egger’s test was performed, and a regression intercept gave a one-tailed *p*-value of 0.029, which also indicated a possible publication bias. Consequently, we conducted a further analysis using Duval and Tweedie’s “Trim and Fill” method and the missing studies were calculated using the random-effects model. The point estimate did not differ significantly when the adjusted values were calculated, indicating that publication bias was not present. The funnel plot with the observed and imputed studies is shown in Figure 7. 

## 4. Discussion

Our meta-analysis included 16 studies with 459 patients who were highly HLA-sensitized, showing favorable grafts and patient outcomes after desensitization with IVIg, plasmapheresis, and rituximab after 1 and 5 years. We also noted a considerable variability in the protocols used by the individual centers for desensitization.

There is significant variability in how class I and class II anti-HLA antibodies influence the incidence of rejection. Combining T and B cell-depleting agents to reduce HLA antibodies, commonly reported as PRA, has been a common strategy for treating individuals who are highly sensitized [38]. The two common IVIg dosing regimens for patients who are HLA-incompatible are a high dose of 2 g/kg and a lower dose of 100 mg/kg [31]. Our meta-analysis included studies using either regimen. There are currently no randomized controlled trials that compare the use of low-dose IVIG and plasmapheresis with high-dose IVIG for desensitization. However, one retrospective study by Stegall et al. compared different desensitization therapies in living-donor transplant recipients with a positive T cell CDC crossmatch. Low-dose IVIG (100 mg/kg) combined with plasmapheresis proved more effective compared to high-dose IVIG (2 g/kg) alone when the T cell CDC crossmatch was positive at intermediate titers (1:8 to 1:16). The chances of effective desensitization decreased when the T cell CDC crossmatch was positive at a high titer (1 > 32) [31]. High doses of IVIG used pre transplant in individuals who are highly sensitized have been associated with certain adverse effects, including aseptic meningitis, thrombotic events, and bronchospasm. Hemolytic anemia has also been observed with IVIG. Chromatographically derived IVIG products contain anti-A and anti-B antibodies in a high titer and can even cause hemolysis [39].

Plasmapheresis removes anti-HLA antibodies, which are immunoglobulin G molecules (IgG) [6]. Numerous studies have reported differences in the occurrence of AbMR after plasmapheresis, which are attributable to the intensity of the circulating DSA. Patients who are CDC crossmatch-positive, therefore, have high incidences of AbMR, followed by other groups, such as high and low fluorescence crossmatch levels [29,40].

In patients treated with rituximab, HLA antibody rebound has been found to occur less frequently and with a lower intensity [9]. Like all immunomodulators, rituximab has been associated with increased infections due to its effects on depleting B cells In highly sensitized, ABO-incompatible renal transplant recipients who received rituximab plus IVIg compared to non-sensitized, ABO-compatible recipients without any pretreatment, the most common infections were bacterial infections (~50%), followed by viral infections (~25%) and fungal infections (~5%), although there was no significant difference found in the two groups [37].]. Also, in a study of renal transplant patients treated with rituximab for various post-transplant conditions, approximately 9% experienced infectious complications [41]. Obinutuzumab is a more efficient anti-CD20 antibody in decreasing the MFI of anti-HLA, but one-third of the patients in a study had severe adverse infectious events [42].

A novel therapy under evaluation is the IgG-degrading enzyme of Streptococcus pyogenes (IdeS), a cysteine endopeptidase which cleaves the lower hinge region separating the F(ab′)2 and Fc fragments. This proteolytic activity prevents complement-mediated injury and antibody-dependent cellular cytotoxicity, thus decreasing the immune response and AbMR. Based on a combined open-label phase I-II trial in the United States and Sweden, 24 out of the 25 recipients who were highly sensitized received successful renal transplants after desensitization with IdeS; one patient had a hyperacute rejection attributed to IgM, IgA, and non-HLA antibodies [43]. Further studies on the efficacy of IdeS are underway, with larger studies and long-term outcome data needed to fully assess its effectiveness.

Although the revamped kidney allocation system in the USA and Europe was designed to alleviate the limited access to deceased donors and decrease the wait times for patients who are highly sensitized, the wait times for patients with high levels of cPRA remain longer compared to patients who are unsensitized [5]. Desensitization effectively addresses the issue of HLA incompatibility and reduces the disparities among recipients awaiting transplantation. Since HLAi is superior to dialysis, these methods offer a solution to decrease the extended wait times [4].

Our study has several limitations. Most of the studies reviewed were retrospective in nature and relied on the accuracy of the data collected, which may have led to gaps in information. It is probable that only a select group of transplant recipients undergo desensitization, suggesting that the results may not be universally applicable. Additionally, heterogeneity was noted in some summary effect sizes. These instances are attributable to the varying PRA levels, different methodologies, timings from desensitization to transplantation, and the presence of other factors affecting graft and patient outcomes. The assay used for CDC crossmatch may vary between different centers. Most of the patients in our meta-analysis underwent living-donor transplantation, and the variables associated with better outcomes of living-donor transplantations were not included. It is possible that less favorable outcomes could be seen in cases of cadaveric-donor transplantations. Also, factors such as hypogammaglobulinemia due to T cell depletion therapy could influence graft and patient survival. Despite having analyzed the potential of publication bias, given that the studies in our meta-analysis are underpowered, some other studies on this topic may have resulted in non-significant results and, consequently, not have been published. Published studies on this topic may disproportionately represent studies with positive findings, and a meta-analysis could result in overestimating the effect sizes. We also reported the rates of UTI, CMV, and BK infections, but further studies are needed to see if these rates differ in patients not undergoing desensitization. Lastly, the donor and recipient factors affecting outcomes such as cold ischemia time, development of new DSA, and compliance rates are different in these studies. Therefore, prudence must be observed while interpreting these results.

To our knowledge, this is the first meta-analysis to assess the outcomes of HLA desensitization using IVIg, plasmapheresis, and rituximab. We report the pooled outcome rates among 459 patients from different countries, which is a strength of this study. In our study, a higher percentage of patients were CDC crossmatch-positive (68.3%), and a majority of them underwent living-donor transplantation (89.4%), with a follow-up period of 41.7 months. Although the different studies used in our analysis were heterogeneous, this study represents the current data available on these patients’ outcomes.

Overall, there were favorable outcomes with desensitization in a select group of transplant recipients, adding valuable data on allograft and patient outcomes. Meticulous patient selection, high-quality care, and ongoing monitoring are crucial for the graft and patient outcomes of patients who are highly HLA-sensitized. Further randomized controlled trials are needed to investigate the effectiveness of desensitization.

## Figures and Tables

**Table 1:** Patient and study characteristics.

Study	Year	Study Period	Country	Total, n	Age (Years), Mean ± SD	Females, n	Dialysis Vintage, in Months, Mean ± SD	Re-Transplant, n	PRA, Mean ± SD	Positive CDC Crossmatch, n	Follow-Up Period (Months), Mean ± SD
Amrouche et al. [2]	2017	2002–2009	France	95	46.6 ± 12.2	51	-	-	-	30	80.3 ± 40.8
Bansal et al. [21]	2021	2014–2018	India	12	39.9 ± 14.4	7	8.8 ± 15.2	0	-	0	26.6 ± 13.9
De Sousa-Amorim et al. [22]	2015	2008–2014	Spain	24	41 ± 13	13	-	18	76 ± 136	1	37 ± 27
Ishida et al. [23]	2021	2011–2020	Japan	41	52 ± 13	22	51.6 ± 25.2	21	-	41	-
Jin et al. [24]	2012	2003–2009	Korea	7	51.4 ± 3.3	6	-	-	41.7 ± 6.1	6	33.2 ± 5.4
Kahwaji et al. [25]	2016	2006–2010	USA	66	-	43	2.73 ± 2.61	33	71 ± 2.32	53	68 ± 15
Keven et al. [26]	2013	-	Turkey	5	40.4 ± 20.9	5	-	2	-	0	17
Loupy et al. [27]	2010	2002–2007	France	18	45 ± 12		-	-	-	7	19.5 ± 9.3
Okada et al. [28]	2018	2012–2015	Japan	15	48.9 ± 13.7	9	48.33 ± 45.9	7	-	15	39.6 ± 12
Reilla et al. [29]	2014	2002–2010	USA	39	43.1 ± 13	27	-	27	47.8 ± 31	39	60
Santos et al. [30]	2014	1999–2013	Portugal	8	42 ± 12	6	168 ± 97.2	6	62 ± 36	-	29.8 ± 35.1
Stegall et al. [31]	2006	2000–2005	USA	61	46.1 ± 16.3	32	-	-	-	-	60
West-Thielke et al. [32]	2008	2001–2007	USA	28	41.9 ± 9.6	21	51.6 ± 67.9	4	66.1 ± 31.8	28	-
Yilmaz et al. [33]	2020	2011–2018	Turkey	16	35 ± 12	8	-	11	53 ± 37	14	39 ± 24
Yoon et al. [34]	2009	2003–2007	Korea	10	47.5 ± 8.4	7	-	3	-	6	52
Zhang et al. [35]	2011	2008–2010	China	14	43.6 ± 7.4	6	-	10	-	-	-

Values are expressed as the mean ± standard deviation or as the number (percentage) of subjects. Abbreviations: CDC—cytotoxicity crossmatch; and PRA—panel-reactive antibody.

**Table 2:** Outcomes of the included studies.

Study	Total, n	IVIg Dose	Plasmapheresis	Rituximab Dose	Controls in the Study	Induction Agent	Post-Transplant Immunosuppression	1-Year Death-Censored Graft Survival, %	5-Year Death-Censored Graft Survival, %	1-Year Patient Survival, %	5-Year Patient Survival, %
Amrouche et al. [2]	95	2 g/kg	5–10 sessions	375 mg/m^2^	39 patients with an MFI between 500 and 3000	Thymoglobulin	Tac + MMF + Prednisone	98%	86%	97%	85%
Bansal et al. [21]	12	100 mg/kg	1–9 sessions	200 mg, 2–3 weeks before transplant	None	Thymoglobulin	Tac + MMF + Prednisone	100%	-	100%	-
De Sousa-Amorim et al. [22]	24	100 mg/kg	1–21 sessions	Two doses of 400 mg, 3–4 weeks before transplantation	None	Thymoglobulin	Tac + MMF + Prednisone	91%	86%	-	95.9%
Ishida et al. [23]	41	2 g/kg	3–4 sessions	200 mg and 300 mg on two separate days	None	NR	Tac + MMF + Prednisone	90%	-	-	-
Jin et al. [24]	7	100 mg/kg	6 sessions	375 mg/m^2^, 2 weeks before transplant	None	Basiliximab	Tac + MMF + Prednisone	100%	100%	100%	100%
Kahwaji et al. [25]	66	2 g/kg	5 sessions	1 gm	111 patients with a low PRA	Alemtuzumab	Tac + MMF + Prednisone	-	90.6%	-	94%
Keven et al. [26]	5	NR	2 sessions	200 mg, one day before transplantation	None	Basiliximab	Tac + MMF + Prednisone	100%	-	100%	-
Loupy et al. [27]	18	2 g/kg	9 sessions	375 mg/m^2^	36 patients received no rituximab	Thymoglobulin	Tac/Cyclosporin + MMF + Prednisone	88.9%	-	94.5%	-
Okada et al. [28]	15	2 g/kg	2–4 sessions	300 mg one month before and 200 mg the day before transplantation	229 crossmatch-ve, DSA -ve	Basiliximab	Tac + MMF + Prednisone	86.7%	-	93.3%	-
Reilla et al. [29]	39	NR	Average of 5.6 sessions	375 mg/m^2^, one day before transplantation	None	Basiliximab (58%), Thymoglobulin (42%)	Tac + MMF + Prednisone	94%	84%	95%	86%
Santos et al. [30]	8	NR	3–9 sessions	375 mg/m^2^	8 patients with a positive flow cytometry crossmatch who were not desensitized	Thymoglobulin	Tac + MMF + Prednisone/Cyclosporine + Azathioprine + Prednisone	100%	71%	88%	-
Stegall et al. [31]	61	100 mg/kg	4–5 sessions	375 mg/m^2^, 4–7 days before transplantation	13 patients high lose IVIg	Thymoglobulin	Tac + MMF + Prednisone	82%	-	93%	-
West-Thielke et al. [32]	28	NR	1–4 sessions	375 mg/m^2^	22 patients who were not African Americans with a positive crossmatch	Thymoglobulin	Tac + MMF + Prednisone	82.6%	-	91%	-
Yilmaz et al. [33]	16	NR	Average of 3.5 sessions	375 mg/m^2^	33 patients received no rituximab	Thymoglobulin	Tac + MMF + Prednisone	93.8%	85.2%	100%	100%
Yoon et al. [34]	10	NR	6 sessions	375 mg/m^2^, one day before transplantation	None	Basiliximab	Tac + MMF + Prednisone	90%	-	100%	-
Zhang et al. [35]	14	100 mg/kg	4–5 sessions	375 mg/m^2^, one week before and one day before transplantation	None	Thymoglobulin	Tac + MMF + Prednisone	92.8%	-	100%	-

Abbreviations: DSA—donor-specific antibodies; IVIg—intravenous immunoglobulin; NR—not reported; MMF—mycofenolate mofetil; and Tac—tacrolimus.

**Table 3:** Summary of pooled rates.

Outcome	Pooled Rate (%)	95% Confidence Interval	Heterogeneity (%)	No. of Studies Reporting the Outcome	Total No. of Patients in the Studies
1-year graft survival	88.9	84.8–92	0	14	377
5-year graft survival	86.1	81.2–89.9	0	7	255
1-year patient survival	94.2	91–96.3	0	13	328
5-year patient survival	88.9	83.5–92.7	7.7	6	247
Antibody-mediated rejection	37.7	25–52.3	80.3	11	375
Acute cell-mediated rejection	15.1	9.1–24	55	10	319
Urinary tract infections	17.1	4.5–47.6	81.5	7	113
Cytomegalovirus infections	8.7	5.2–14.1	0	8	185
BK nephropathy	10	6–16.2	0	6	149

**Table 4:** Quality assessment of the studies using the Newcastle—Ottawa Quality Assessment Form.

Study	Representativeness of the Average Adult in Community	Cohort Size	Information on Outcomes	Outcome Not Present at Start	Additional Intervention	Adequate Assessment	Follow-Up Time	Adequacy of Follow-Up	Max 7, High > 5, Medium 3–5, Low <3
	Population-Based: 1; Multi-Center: 0.5; Single-Center: 0	>40 Patients: 1; 39 to 20: 0.5; <20: 0	Information with Clarity: 1; Information Derived: 0.5	Not Present: 1; Present: 0	Yes: 1; No: 0	Yes: 1; No: 0	Yes: 1; Not Mentioned: 0	All Patients Followed-Up: 1; >50% Followed-Up: 0.5; <50% Followed-Up OR Not Mentioned: 0	
Amrouche et al. [2]	0	1	1	0	1	1	1	1	6
Bansal et al. [21]	0	0	1	0	1	1	1	1	5
De Sousa-Amorim et al. [22]	0	0.5	1	1	1	1	1	1	6.5
Ishida et al. [23]	0	1	1	1	1	1	1	0	6
Jin et al. [24]	0	0	1	0	1	1	1	1	5
Kahwaji et al. [25]	0	1	1	0	1	1	1	1	6
Keven et al. [26]	0	0	1	0	1	0	1	1	4
Loupy et al. [27]	0	0	1	0	1	1	1	1	5
Okada et al. [28]	0	0	1	0	1	1	1	1	5
Reilla et al. [29]	0	0.5	1	0	1	1	1	1	5.5
Santos et al. [30]	0	0	0.5	1	1	0	1	1	4.5
Stegall et al. [31]	0	1	0.5	1	1	1	1	1	6
West-Thielke et al. [32]	0	0.5	1	1	1	1	1	1	6.5
Yilmaz et al. [33]	0	0	0.5	1	1	0	1	1	4.5
Yoon et al. [34]	0	0	0.5	1	1	0	1	1	4.5
Zhang et al. [35]	0	0	0.5	1	1	0	1	1	4.5

## Data Availability

The data used in the study are available and will be shared upon reasonable request.

## Supplementary Materials

The following supporting information can be downloaded at https://www.mdpi.com/article/10.3390/life14080998/s1: Table S1: The Query and the search results for each database, Table S2: MOOSE Checklist for Meta-analyses of Observational Studies [44] and Table S3: Preferred Reporting Items for Systematic Reviews and Meta-Analyses: The PRISMA Statement [45]. References [44,45] are cited in the supplementary materials.

## References

[1] Sethi S., Choi J., Toyoda M., Vo A., Peng A., Jordan S.C. (2017). Desensitization: Overcoming the Immunologic Barriers to Transplantation. J. Immunol. Res..

[2] Amrouche L., Aubert O., Suberbielle C., Rabant M., Van Huyen J.D., Martinez F., Sberro-Soussan R., Scemla A., Tinel C., Snanoudj R. (2017). Long-term Outcomes of Kidney Transplantation in Patients with High Levels of Preformed DSA: The Necker High-Risk Transplant Program. Transplantation.

[3] Mamode N., Bestard O., Claas F., Furian L., Griffin S., Legendre C., Pengel L., Naesens M. (2022). European Guideline for the Management of Kidney Transplant Patients with HLA Antibodies: By the European Society for Organ Transplantation Working Group. Transpl. Int..

[4] Orandi B.J., Luo X., Massie A.B., Garonzik-Wang J.M., Lonze B.E., Ahmed R., Van Arendonk K.J., Stegall M.D., Jordan S.C., Oberholzer J. (2016). Survival Benefit with Kidney Transplants from HLA-Incompatible Live Donors. N. Engl. J. Med..

[5] Schinstock C.A., Smith B.H., Montgomery R.A., Jordan S.C., Bentall A.J., Mai M., Khamash H.A., Stegall M.D. (2019). Managing highly sensitized renal transplant candidates in the era of kidney paired donation and the new kidney allocation system: Is there still a role for desensitization?. Clin. Transplant..

[6] Noble J., Jouve T., Malvezzi P., Rostaing L. (2023). Desensitization in Crossmatch-positive Kidney Transplant Candidates. Transplantation.

[7] Kazatchkine M.D., Kaveri S.V. (2001). Immunomodulation of autoimmune and inflammatory diseases with intravenous immune globulin. N. Engl. J. Med..

[8] Schwab I., Nimmerjahn F. (2013). Intravenous immunoglobulin therapy: How does IgG modulate the immune system?. Nat. Rev. Immunol..

[9] Jackson A.M., Kraus E.S., Orandi B.J., Segev D.L., Montgomery R.A., Zachary A.A. (2015). A closer look at rituximab induction on HLA antibody rebound following HLA-incompatible kidney transplantation. Kidney Int..

[10] Page M.J., McKenzie J.E., Bossuyt P.M., Boutron I., Hoffmann T.C., Mulrow C.D., Shamseer L., Tetzlaff J.M., Akl E.A., Brennan S.E. (2021). The PRISMA 2020 statement: An updated guideline for reporting systematic reviews. Int. J. Surg..

[11] Borenstein M., Hedges L.V., Higgins J.P., Rothstein H.R. (2010). A basic introduction to fixed-effect and random-effects models for meta-analysis. Res. Synth. Methods.

[12] Barker T.H., Migliavaca C.B., Stein C., Colpani V., Falavigna M., Aromataris E., Munn Z. (2021). Conducting proportional meta-analysis in different types of systematic reviews: A guide for synthesisers of evidence. BMC Med. Res. Methodol..

[13] DerSimonian R., Laird N. (1986). Meta-analysis in clinical trials. Control. Clin. Trials.

[14] Sutton A.J., Abrams K.R., Jones D.R., Jones D.R., Sheldon T.A., Song F. (2000). Methods for Meta-Analysis in Medical Research.

[15] Higgins J.P., Thompson S.G., Deeks J.J., Altman D.G. (2003). Measuring inconsistency in meta-analyses. BMJ.

[16] Duval S., Tweedie R. (2000). Trim and fill: A simple funnel-plot–based method of testing and adjusting for publication bias in meta-analysis. Biometrics.

[17] Easterbrook P.J., Gopalan R., Berlin J., Matthews D.R. (1991). Publication bias in clinical research. Lancet.

[18] Comprehensive Meta-Analysis Software, version 4; 2022. https://meta-analysis.com/pages/full.

[19] Wells G.A., Shea B., O’Connell D., Peterson J., Welch V., Losos M., Tugwell P. The Newcastle-Ottawa Scale (NOS) for Assessing the Quality of Nonrandomised Studies in Meta-Analyses. Proceedings of the 3rd Symposium on Systematic Reviews: Beyond the Basics.

[20] Clark J., Glasziou P., Del Mar C., Bannach-Brown A., Stehlik P., Scott A.M. (2020). A full systematic review was completed in 2 weeks using automation tools: A case study. J. Clin. Epidemiol..

[21] Bansal S.B., Gade A., Sinha S., Mahapatra A., Jha P., Sethi S.K. (2021). HLA Desensitization Based on Results of the Luminex Technique in Kidney Transplant—A Single-center Experience. Indian J. Nephrol..

[22] De Sousa-Amorim E., Revuelta I., Blasco M., Diekmann F., Cid J., Lozano M., Sánchez-Escuredo A., Martorell J., Palou E., Campistol J. (2015). Desensitization Before Living Donor Kidney Transplantation in Highly HLA-Sensitized Patients: A Single-Center Study. Transplant. Proc..

[23] Ishida H., Unagami K., Omoto K., Kanzawa T., Tanabe K. (2021). Desensitization Regimen Consisting of High-Dose Intravenous Immunoglobulin, Plasmapheresis, and Rituximab (an Anti-CD20 Antibody), Without Eculizumab and/or Bortezomib, in 41 Highly Sensitized Kidney Transplant Recipients. Exp. Clin. Transplant. Off. J. Middle East Soc. Organ Transplant..

[24] Jin M.K., Cho J.H., Kwon O., Hong K.D., Choi J.Y., Yoon S.H., Park S.-H., Kim Y.-L., Kim C.-D. (2012). Successful kidney transplantation after desensitization using plasmapheresis, low-dose intravenous immunoglobulin, and rituximab in highly sensitized patients: A single-center experience. Transplant. Proc..

[25] Kahwaji J., Jordan S.C., Najjar R., Wongsaroj P., Choi J., Peng A., Villicana R., Vo A. (2016). Six-year outcomes in broadly HLA-sensitized living donor transplant recipients desensitized with intravenous immunoglobulin and rituximab. Transpl. Int..

[26] Keven K., Sengul S., Celebi Z.K., Tuzuner A., Yalcin F., Duman T., Tutkak H. (2013). Kidney transplantation in immunologically high-risk patients. Transplant. Proc..

[27] Loupy A., Suberbielle-Boissel C., Zuber J., Anglicheau D., Timsit M.O., Martinez F., Thervet E., Bruneval P., Charron D., Hill G.S. (2010). Combined posttransplant prophylactic IVIg/anti-CD 20/plasmapheresis in kidney recipients with preformed donor-specific antibodies: A pilot study. Transplantation.

[28] Okada D., Okumi M., Kakuta Y., Unagami K., Iizuka J., Takagi T., Ishida H., Tanabe K. (2018). Outcome of the risk-stratified desensitization protocol in donor-specific antibody-positive living kidney transplant recipients: A retrospective study. Transpl. Int..

[29] Riella L.V., Safa K., Yagan J., Lee B., Azzi J., Najafian N., Abdi R., Milford E., Mah H., Gabardi S. (2014). Long-term outcomes of kidney transplantation across a positive complement-dependent cytotoxicity crossmatch. Transplantation.

[30] Santos C., Costa R., Malheiro J., Pedroso S., Almeida M., Martins L., Dias L., Tafulo S., Henriques A., Cabrita A. (2014). Kidney transplantation across a positive crossmatch: A single-center experience. Transplantation Proceedings.

[31] Stegall M.D., Gloor J., Winters J.L., Moore S.B., Degoey S. (2006). A comparison of plasmapheresis versus high-dose IVIG desensitization in renal allograft recipients with high levels of donor specific alloantibody. Am. J. Transplant..

[32] West-Thielke P., Herren H., Thielke J., Oberholzer J., Sankary H., Raofi V., Benedetti E., Kaplan B. (2008). Results of positive cross-match transplantation in African American renal transplant recipients. Am. J. Transplant..

[33] Yilmaz V.T., Kisaoglu A., Dandin O., Demiryilmaz I., Koksoy S., Aydinli B., Kocak H. (2020). Living donor kidney transplantation after desensitization in cross-match positive high sensitized patients. Hippokratia.

[34] Yoon H.E., Hyoung B.J., Hwang H.S., Lee S.Y., Jeon Y.J., Song J.C., Oh E.-J., Park S.C., Choi B.S., Moon I.S. (2009). Successful renal transplantation with desensitization in highly sensitized patients: A single center experience. J. Korean Med. Sci..

[35] Zhang W., Chen D., Chen Z., Zeng F., Ming C., Lin Z., Zhou P., Chen G., Chen X. (2011). Successful kidney transplantation in highly sensitized patients. Front. Med..

[36] Ishida H., Furusawa M., Shimizu T., Nozaki T., Tanabe K. (2014). Influence of preoperative anti-HLA antibodies on short- and long-term graft survival in recipients with or without rituximab treatment. Transpl. Int..

[37] Kahwaji J., Sinha A., Toyoda M., Ge S., Reinsmoen N., Cao K., Lai C.H., Villicana R., Peng A., Jordan S. (2011). Infectious complications in kidney-transplant recipients desensitized with rituximab and intravenous immunoglobulin. Clin. J. Am. Soc. Nephrol..

[38] Montgomery R.A., Lonze B.E., King K.E., Kraus E.S., Kucirka L.M., Locke J.E., Warren D.S., Simpkins C.E., Dagher N., Singer A.L. (2011). Desensitization in HLA-incompatible kidney recipients and survival. N. Engl. J. Med..

[39] Jordan S.C., Toyoda M., Kahwaji J., Vo A.A. (2011). Clinical aspects of intravenous immunoglobulin use in solid organ transplant recipients. Am. J. Transplant..

[40] Gloor J., Winters J., Cornell L., Fix L., DeGoey S., Knauer R., Cosio F.G., Gandhi M.J., Kremers W., Stegall M.D. (2010). Baseline donor-specific antibody levels and outcomes in positive crossmatch kidney transplantation. Am. J. Transplant..

[41] Kamar N., Milioto O., Puissant-Lubrano B., Esposito L., Pierre M.C., Mohamed A.O., Lavayssière L., Cointault O., Ribes D., Cardeau I. (2010). Incidence and predictive factors for infectious disease after rituximab therapy in kidney-transplant patients. Am. J. Transplant..

[42] Redfield R.R., Jordan S.C., Busque S., Vincenti F., Woodle E.S., Desai N., Reed E.F., Tremblay S., Zachary A.A., Vo A.A. (2019). Safety, pharmacokinetics, and pharmacodynamic activity of obinutuzumab, a type 2 anti-CD20 monoclonal antibody for the desensitization of candidates for renal transplant. Am. J. Transplant..

[43] Jordan S.C., Lorant T., Choi J., Kjellman C., Winstedt L., Bengtsson M., Zhang X., Eich T., Toyoda M., Eriksson B.M. (2017). IgG Endopeptidase in Highly Sensitized Patients Undergoing Transplantation. N. Engl. J. Med..

[44] Observational Studies in Epidemiology (MOOSE) Group (2000). Meta-analysis of Observational Studies in Epidemiology. A Proposal for Reporting. JAMA.

[45] Moher D., Liberati A., Tetzlaff J., Altman D.G. (2009). The PRISMA Group. Preferred Reporting Items for Systematic Reviews and Meta-Analyses: The PRISMA Statement. PLoS Med..

