# Supplementary material for: Outcomes of Kidney Transplantation in Highly HLA-Sensitized Patients Treated with Intravenous Immuno-Globulin, Plasmapheresis and Rituximab: A Meta-Analysis
Source: Life (Basel). 2024 Aug 10;14(8):998. doi: 10.3390/life14080998 (PMC11355159; doi:10.3390/life14080998)
Supplement: Supplementary file 1 [file life-14-00998-s001.zip › life-3127255-supplementary.pdf]

Supplementary information

Literature search

A systematic literature review was conducted to evaluate the contemporary literature on HLA desensitization utilizing IVIg, plasmapheresis and rituximab.

Documentary sources consulted

We carried out a comprehensive literature review using the Google scholar (via Harzing's Publish or Perish 8), PubMed (https://pubmed.ncbi.nlm.nih.gov/, National Library of Medicine, National Institutes of Health), Embase (Embase.com, Elsevier), Web of Science (Core Collection, Clarivate) and Google scholar databases. Systematic Review Accelerator's Deduplicator was used to identify duplicates.

Search strategy

For a comprehensive search suitable MeSH (Medical Subject Headings) and Index terms were identified to be included in the search term. Index terms and MeSH terms are controlled vocabularies used to standardize literature indexing, allowing systematic and accurate retrieval of articles from databases.

MeSH (Medical Subject Headings): "HLA desensitization" OR "hla antigens" OR "histocompatibility antigens class i" OR "histocompatibility antigens class ii" OR "immunologic desensitization" OR "desensitize" AND "kidney transplantation" OR "renal transplantation" OR "treatment".

Table S1. The Query and the search results for each database.

PubMed (Searched on 6/9/2023

("HLA desensitization"[All Fields] OR (("hla antigens"[MeSH Terms] OR ("hla"[All Fields] AND "antigens"[All Fields]) OR "hla antigens"[All Fields] OR ("human"[All Fields] AND "leukocyte"[All Fields] AND "antigen"[All Fields]) OR "human leukocyte antigen"[All Fields] OR "histocompatibility antigens class ii"[MeSH Terms] OR ("histocompatibility"[All Fields] AND "antigens"[All Fields] AND "class"[All Fields] AND "ii"[All Fields]) OR "histocompatibility antigens class ii"[All Fields] OR ("human"[All Fields] AND "leukocyte"[All Fields] AND "antigen"[All Fields]) OR "human leukocyte antigen"[All Fields] OR	265

"histocompatibility antigens class i"[MeSH Terms] OR "histocompatibility antigens class i"[All Fields] OR ("human"[All Fields] AND "leukocyte"[All Fields] AND "antigen"[All Fields])) AND ("desensitise"[All Fields] OR "desensitised"[All Fields] OR "desensitiser"[All Fields] OR "desensitises"[All Fields] OR "desensitising"[All Fields] OR "desensitization, immunologic"[MeSH Terms] OR ("desensitization"[All Fields] AND "immunologic"[All Fields]) OR "immunologic desensitization"[All Fields] OR "desensitisation"[All Fields] OR "desensitization"[All Fields] OR "desensitizations"[All Fields] OR "desensitize"[All Fields] OR "desensitized"[All Fields] OR "desensitizer"[All Fields] OR "desensitizers"[All Fields] OR "desensitizes"[All Fields] OR "desensitizing"[All Fields]))) AND ("kidney transplantation"[All Fields] OR "renal transplantation"[All Fields]) AND ("therapy"[All Fields] OR "treatment"[All Fields] OR "intervention"[All Fields])	

No limits or filters.

Embase (Embase.com, Elsevier) Searched on 6/9/2023

('hla sensitization' OR ((hla AND ('antigen'/exp OR antigens) OR 'hla antigen'/exp OR 'hla antigens' OR (('histocompatibility'/exp OR histocompatibility) AND ('antigen'/exp OR antigens) AND class AND 2) OR 'hla antigen class 2'/exp OR 'histocompatibility antigens class ii' OR 'human leukocyte antigen'/exp OR 'human leukocyte antigen' OR 'hla antigen class 1'/exp OR 'histocompatibility antigens class i' OR (('human'/exp OR human) AND ('leukocyte'/exp OR leukocyte) AND ('antigen'/exp OR antigen))) AND (desensitise OR desensitised OR desensitiser OR desensitises OR desensitising OR (('desensitization'/exp OR desensitization) AND immunologic) OR 'immunologic desensitization' OR 'desensitisation'/exp OR desensitisation OR 'desensitization'/exp OR desensitization OR desensitizations OR desensitize OR desensitized OR desensitizer OR desensitizers OR desensitizes OR desensitizing))) AND ('kidney transplantation'/exp OR 'kidney transplantation' OR 'renal transplantation') AND ('therapy'/exp OR therapy OR 'treatment'/exp OR treatment OR 'intervention'/exp OR intervention)	883

No limits or filters.

Web of Science (Core Collection, Clarivate) searched on 6/9/2023

**ALL=(("HLA desensitization" OR (("hla antigens" OR (hla AND antigens) OR "hla antigens" OR (human AND leukocyte AND antigen) OR "human leukocyte antigen" OR "histocompatibility antigens class ii" OR (histocompatibility AND antigens AND class AND ii) OR "histocompatibility antigens class ii" OR (human AND leukocyte AND antigen) OR "human leukocyte antigen" OR "histocompatibility antigens class i" OR "histocompatibility antigens class i" OR (human AND leukocyte AND**	169

**antigen)) AND (desensitise OR desensitised OR desensitiser OR desensitises OR desensitising OR "desensitization, immunologic" OR (desensitization AND immunologic) OR "immunologic desensitization" OR desensitisation OR desensitization OR desensitizations OR desensitize OR desensitized OR desensitizer OR desensitizers OR desensitizes OR desensitizing))) AND ("kidney transplantation" OR "renal transplantation") AND (therapy OR treatment OR intervention))**	

No limits or filters.

Google Scholar via Harzing's Publish or Perish on 6/9/2023

("HLA desensitization" OR "HLA Antigens" OR "histocompatibility antigens" OR "human leukocyte antigen") AND (desensitization OR desensitize) AND ((kidney OR renal) AND (transplant OR transplantation))	200

Limited to 200 citations

Table S2. MOOSE Checklist for Meta-analyses of Observational Studies

Item No	Recommendation	Reported on Page No
Reporting of background should include		
1	Problem definition	3
2	Hypothesis statement	-
3	Description of study outcome(s)	6
4	Type of exposure or intervention used	6
5	Type of study designs used	7
6	Study population	6
Reporting of search strategy should include		
7	Qualifications of searchers (eg, librarians and investigators)	Title page,
8	Search strategy, including time period included in the synthesis and key words	5, Table 1
9	Effort to include all available studies, including contact with authors	5,6
10	Databases and registries searched	5
11	Search software used, name and version, including special features used (eg, explosion)	5, Supplementary data
12	Use of hand searching (eg, reference lists of obtained articles)	5
13	List of citations located and those excluded, including justification	8, Fig 1
14	Method of addressing articles published in languages other than English	Figure 1
15	Method of handling abstracts and unpublished studies	6, Figure 1
16	Description of any contact with authors	-
Reporting of methods should include		
17	Description of relevance or appropriateness of studies assembled for assessing the hypothesis to be tested	8, 9
18	Rationale for the selection and coding of data (eg, sound clinical principles or convenience)	8
19	Documentation of how data were classified and coded (eg, multiple raters, blinding and interrater reliability)	8, 10
20	Assessment of confounding (eg, comparability of cases and controls in studies where appropriate)	10, Table 3
21	Assessment of study quality, including blinding of quality assessors, stratification or regression on possible predictors of study results	10, Table 3
22	Assessment of heterogeneity	10, 11, Table 4
23	Description of statistical methods (eg, complete description of fixed or random effects models, justification of whether the chosen models account for predictors of study results, dose-response models, or cumulative meta-analysis) in sufficient detail to be replicated	7
24	Provision of appropriate tables and graphics	Tables 1-4, Figs 1-7

Reporting of results should include		
25	Graphic summarizing individual study estimates and overall estimate	Figs 2-6
26	Table giving descriptive information for each study included	Table 1, 2
27	Results of sensitivity testing (eg, subgroup analysis)	Fig 6
28	Indication of statistical uncertainty of findings	14

Item No	Recommendation	Reported on Page No
Reporting of discussion should include		
29	Quantitative assessment of bias (eg, publication bias)	11, Fig 7
30	Justification for exclusion (eg, exclusion of non-English language citations)	Figure 1
31	Assessment of quality of included studies	10, Table 3
Reporting of conclusions should include		
32	Consideration of alternative explanations for observed results	13, 14
33	Generalization of the conclusions (ie, appropriate for the data presented and within the domain of the literature review)	14
34	Guidelines for future research	14
35	Disclosure of funding source	15

From: Stroup DF, Berlin JA, Morton SC, et al, for the Meta-analysis Of Observational Studies in Epidemiology (MOOSE) Group. Meta-analysis of Observational Studies in Epidemiology. A Proposal for Reporting. *JAMA*. 2000;283(15):2008-2012. doi: 10.1001/jama.283.15.2008. [44]

Table S3. Preferred Reporting Items for Systematic Reviews and Meta-Analyses: The PRISMA Statement

Section/topic	#	Checklist item	Reported on page #
**TITLE**			
Title	1	Identify the report as a systematic review, meta-analysis, or both.	1
**ABSTRACT**			
Structured summary	2	Provide a structured summary including, as applicable: background; objectives; data sources; study eligibility criteria, participants, and interventions; study appraisal and synthesis methods; results; limitations; conclusions and implications of key findings; systematic review registration number.	2
**INTRODUCTION**			
Rationale	3	Describe the rationale for the review in the context of what is already known.	3,4
Objectives	4	Provide an explicit statement of questions being addressed with reference to participants, interventions, comparisons, outcomes, and study design (PICOS).	4
**METHODS**			
Protocol and registration	5	Indicate if a review protocol exists, if and where it can be accessed (e.g., Web address), and, if available, provide registration information including registration number.	6
Eligibility criteria	6	Specify study characteristics (e.g., PICOS, length of follow-up) and report characteristics (e.g., years considered, language, publication status) used as criteria for eligibility, giving rationale.	6
Information sources	7	Describe all information sources (e.g., databases with dates of coverage, contact with study authors to identify additional studies) in the search and date last searched.	5
Search	8	Present full electronic search strategy for at least one database, including any limits used, such that it could be repeated.	5, Figure 1, Supplementary information
Study selection	9	State the process for selecting studies (i.e., screening, eligibility, included in systematic review, and, if applicable, included in the meta-analysis).	6
Data collection process	10	Describe method of data extraction from reports (e.g., piloted forms, independently, in duplicate) and any processes for obtaining and confirming data from investigators.	6
Data items	11	List and define all variables for which data were sought (e.g., PICOS, funding sources) and any assumptions and simplifications made.	6
Risk of bias in individual studies	12	Describe methods used for assessing risk of bias of individual studies (including specification of whether this was done at the study or outcome level), and how this information is to be used	7,8

		in any data synthesis.	
Summary measures	13	State the principal summary measures (e.g., risk ratio, difference in means).	7
Synthesis of results	14	Describe the methods of handling data and combining results of studies, if done, including measures of consistency (e.g., I^2) for each meta-analysis.	7
Risk of bias across studies	15	Specify any assessment of risk of bias that may affect the cumulative evidence (e.g., publication bias, selective reporting within studies).	7,8
Additional analyses	16	Describe methods of additional analyses (e.g., sensitivity or subgroup analyses, meta-regression), if done, indicating which were pre-specified.	11, Figure 6
**RESULTS**			
Study selection	17	Give numbers of studies screened, assessed for eligibility, and included in the review, with reasons for exclusions at each stage, ideally with a flow diagram.	8
Study characteristics	18	For each study, present characteristics for which data were extracted (e.g., study size, PICOS, follow-up period) and provide the citations.	8,9
Risk of bias within studies	19	Present data on risk of bias of each study and, if available, any outcome level assessment (see item 12).	10
Results of individual studies	20	For all outcomes considered (benefits or harms), present, for each study: (a) simple summary data for each intervention group (b) effect estimates and confidence intervals, ideally with a forest plot.	10, Figure 2-5
Synthesis of results	21	Present the main results of the review. If meta-analyses are done, include for each, confidence intervals and measures of consistency	10
Risk of bias across studies	22	Present results of any assessment of risk of bias across studies (see Item 15).	10, Table 3
Additional analysis	23	Give results of additional analyses, if done (e.g., sensitivity or subgroup analyses, meta-regression [see Item 16]).	11, Figure 6
**DISCUSSION**			
Summary of evidence	24	Summarize the main findings including the strength of evidence for each main outcome; consider their relevance to key groups (e.g., healthcare providers, users, and policy makers).	11, 12
Limitations	25	Discuss limitations at study and outcome level (e.g., risk of bias), and at review-level (e.g., incomplete retrieval of identified research, reporting bias).	13, 14

Conclusions	26	Provide a general interpretation of the results in the context of other evidence, and implications for future research.	14
**FUNDING**			
Funding	27	Describe sources of funding for the systematic review and other support (e.g., supply of data); role of funders for the systematic review.	15

From: Moher D, Liberati A, Tetzlaff J, Altman DG, The PRISMA Group (2009). Preferred Reporting Items for Systematic Reviews and Meta-Analyses: The PRISMA Statement. PLoS Med 6(7): e1000097. doi:10.1371/journal.pmed1000097

For more information, visit: **www.prisma-statement.org**. [45]
